# Abdominal tissue concentrations and penetration of carboplatin in a HIPEC procedure ‒ assessment in a novel porcine model

**DOI:** 10.1515/pp-2022-0110

**Published:** 2022-06-06

**Authors:** Elisabeth K. Petersen, Mats Bue, Christina Harlev, Andrea R. Jørgensen, Anne Schmedes, Pelle Hanberg, Lone K. Petersen, Maiken Stilling

**Affiliations:** Department of Orthopaedic Surgery, Aarhus University Hospital, Aarhus, Denmark; Department of Clinical Medicine, Aarhus University Hospital, Aarhus, Denmark; Department of Biochemistry and Immunology, Lillebaelt Hospital, Vejle, Denmark; Department of Gynaecology and Obstetrics, Odense University Hospital and Department of Clinical Medicine, University of Southern Denmark, Odense, Denmark

**Keywords:** carboplatin, hyperthermic intraperitoneal chemotherapy, microdialysis, pharmacokinetics

## Abstract

**Objectives:**

Peritoneal dissemination from intraabdominal cancers is associated with poor prognosis and rapid disease progression. Hyperthermic intraperitoneal chemotherapy (HIPEC) is an antineoplastic treatment, which has improved survival and recurrence-free survival, but little is known about the acquired chemotherapy concentrations in local tissues. The aim of this study was to assess concentrations of carboplatin during and after HIPEC treatment dynamically and simultaneously in various abdominal organ tissues by means of microdialysis in a novel porcine model.

**Methods:**

Eight pigs underwent imitation cytoreductive surgery followed by HIPEC (90 min) using a carboplatin dosage of 800 mg/m^2^. Microdialysis catheters were placed for sampling of drug concentrations in various solid tissues: peritoneum, liver, bladder wall, mesentery and in different depths of one mm and four mm in the hepatoduodenal ligament and rectum. During and after HIPEC, dialysates and blood samples were collected over 8 h.

**Results:**

No statistically significant differences in mean AUC_0-last_ (range: 2,657–5,176 min·µg/mL), mean C_max_ (range: 10.6–26.0 µg/mL) and mean T_max_ (range: 105–206 min) were found between the compartments. In plasma there was a tendency towards lower measures. No difference between compartments was found for tissue penetration. At the last samples obtained (450 min) the mean carboplatin concentrations were 4.9–9.9 µg/mL across the investigated solid tissues.

**Conclusions:**

Equal carboplatin distribution in abdominal organ tissues, detectable concentrations for at least 6 h after HIPEC completion, and a carboplatin penetration depth of minimum four mm were found. The present study proposes a new HIPEC porcine model for future research.

## Introduction

Cancers within the abdominal cavity, including gynaecological cancers and colorectal cancer, are often diagnosed in a late stage. In these cases, carcinomatosis advancement is associated with poor prognosis and high morbidity despite the use of systemic antineoplastic treatment [1]. Local intraperitoneal chemotherapy was introduced in the 1950s and subsequent combined with cytoreductive surgery (CRS), defined as removal of visible tumours, as a treatment option for advanced carcinomatosis. This changed the palliative approach to treatment with a curative intent for a higher number of patients [2].

Hyperthermic intraperitoneal chemotherapy (HIPEC) is a refinement of the intraperitoneal chemotherapy treatment performed after CRS. In HIPEC, the chemotherapeutic solution is circulated intraperitoneal and heated to 40–43 °C as the hyperthermia increases chemotherapeutic cytotoxicity [3]. For ovarian cancer stage III with peritoneal carcinomatosis, CRS combined with HIPEC treatment compared to CRS alone has been associated with an increased recurrence-free survival of at least three months and an increased overall survival by approximately one year [4, 5].

Although HIPEC has been used during the last decades, its application remains empirical, and current literature, regarding local tissue concentrations and penetration depth of the applied chemotherapy, is limited. Few experimental rodent studies have investigated the penetration depth and tissue concentrations of intraperitoneally bolus-injected chemotherapy in tissue and tumour biopsies [6], [7], [8]. However, the biopsy method is limited by its invasiveness, static sampling points, determination of total and not free drug concentration, and by its potential risk of sample contamination from the surroundings [9]. To overcome these limitations, microdialysis has evolved as a promising catheter-based method allowing for continuous and simultaneous sampling of the free drug concentrations from multiple target tissues with a high temporal resolution [10]. This enables investigation of chemotherapy concentrations in local abdominal target tissues and penetration depth throughout and after HIPEC. To our knowledge microdialysis has not previously been applied in HIPEC research.

Carboplatin is a chemotherapeutic agent widely used in the systemic treatment following surgery for ovarian cancer, lung cancer and testicle cancer. It is associated with less nephro- and gastro-intestinal toxicity than other frequently used platinum compounds, e.g. cisplatin [11], [12], [13]. The pharmacokinetic profile for carboplatin has been sparsely studied and only investigated in plasma and the intraperitoneal perfusate during HIPEC [14, 15].

This study aimed (1) to test the feasibility of using microdialysis for dynamic sampling of carboplatin concentrations simultaneously from various abdominal organ tissues during and after HIPEC in a novel large porcine model, and (2) to assess carboplatin concentrations at different tissue depths.

### Potential clinical relevance

#### What is already known

Cytoreductive surgery combined with HIPEC is known to improve the prognosis for patients with abdominal cancers with peritoneal dissemination, but the use of HIPEC is based on empirical knowledge.

#### What is new

A relevant preclinical model has been warranted for systematic HIPEC research. This study presents a novel porcine model for HIPEC procedure with carboplatin.

#### Potential impact

With this model it will be possible to study HIPEC parameters such as temperature, treatment time duration and chemotherapeutic drug dosage etc. for future improvements of HIPEC.

## Materials and methods

The study was conducted at the Institute of Clinical Medicine, Aarhus University Hospital, Denmark. The chemical analyses were performed at Lillebaelt Hospital, Vejle, Denmark. The study was approved by the Danish Animal Experiments Inspectorate (licence No. 2017/15-0201-01184) and was carried out according to existing laws and institutional policies.

### Overview

**Figure 1:**
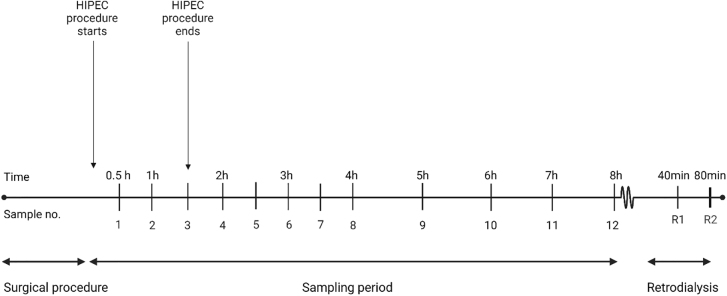
Timeline illustrating the sampling process. Time is shown above the timeline and sample numbers are shown under the timeline. Recovery samples from the calibration period are named R1 and R2 (Created with BioRender.com).

Eight female pigs (Danish Landrace Breed, weight 71–83 kg, age; five months) were included in the study, which underwent an imitation of CRS followed by HIPEC. Carboplatin concentrations were obtained with microdialysis from various peritoneal locations and intrabdominal organs (Table 1). Sampling was conducted for 8 h starting from the initiation of the HIPEC procedure with carboplatin 800 mg/m^2^ (Figure 1) [15, 16].

**Table 1:** Placement of microdialysis catheters in the investigated solid tissues within the abdomen.

Organs/tissue	Number of microdialysis catheters	Depth placement of microdialysis catheters
Liver	1	
Hepatoduodenal ligament	2	1 mm (superficial) and 4 mm (profound)
Peritoneum, right abdominal wall	1	
Rectum	2	1 mm (superficial) and 4 mm (profound)
Bladder wall	1	
Mesentery	1	
Total	8	

MD, microdialysis.

### Microdialysis

Microdialysis, as a sampling tool, consists of a catheter connected to a perfusion pump. The method is based on passive diffusion along the concentration gradient across a semipermeable membrane at the tip of the microdialysis catheter. Due to continuous perfusion through the microdialysis system, dialysates can continuously be obtained, representing the free drug concentrations [17]. However, with constant perfusion of the microdialysis catheter, total equilibrium will never be reached, and the drug concentration in the dialysate is only a fraction of the true drug concentration in the fluid or tissue surrounding the probe. This fraction is referred to as the relative recovery (RR) [18]. Therefore, individual catheter calibration must be performed to obtain absolute tissue concentrations. In this study, calibration was conducted by the retrodialysis-by-drug method, performed after the sampling period (Figure 1) [18, 19]. RR was calculated with the following equation:

$$\text{RR}=100\cdot \left(1-\frac{{\text{C}}_{\text{dialysate}}}{{\text{C}}_{\text{perfusate}}}\right)$$

C_dialysate_ is the concentration of carboplatin in the dialysate (µg/mL), and C_perfusate_ is the concentration of carboplatin in the perfusate (µg/mL). The individual catheter calibration was performed using a C_perfusate_ carboplatin concentration of 100 µg/mL and applying the mean value of two 40 min samples.

The absolute carboplatin concentrations, C_tissue_, were calculated by RR-correction using the following equation:

$$C_{\text{tissue}}=100\cdot \frac{C_{\text{dialysate}}}{RR}$$

All measured concentrations were applied to the midpoint of the sampling interval. A more in-depth description of microdialysis can be found elsewhere [19, 20].

Microdialysis equipment from M Dialysis AB (Stockholm, Sweden) was utilized, comprising of 63 microdialysis catheters (membrane length: 30 mm; molecular cut off: 20 kilo-Dalton), and microdialysis 107 pumps, inducing a flow rate of 1 µL/min with 0.9% NaCl.

### Anaesthesia and surgical procedures

During the experiment the pigs were kept under general anaesthesia with a continuous infusion of propofol (400–600 mg/h, Fresenius Kabi, Bad Homburg, Germany) and fentanyl (1.2–1.5 mg/h, B. Braun, Melsungen, Germany). Arterial pH was kept within 7.3–7.5. Core temperature was measured rectally and kept within 36.5–40.0 °C. All pigs were euthanized at the end of the experiment by of intra-venous administered pentobarbital.

With the animal in a supine position a surgical procedure imitating CRS was performed: Via a midline incision from the symphysis to the xiphoid process the ovaries and uterus were presented and bilateral salpingooforectomy and hysterectomy were performed. Next, the omentum was presented and total omentectomy was performed together with peritoneal stripping with removal of a 10 × 10 cm peritoneum from the left anterior abdominal wall to imitate removal of peritoneal metastasis.

**Figure 2:**
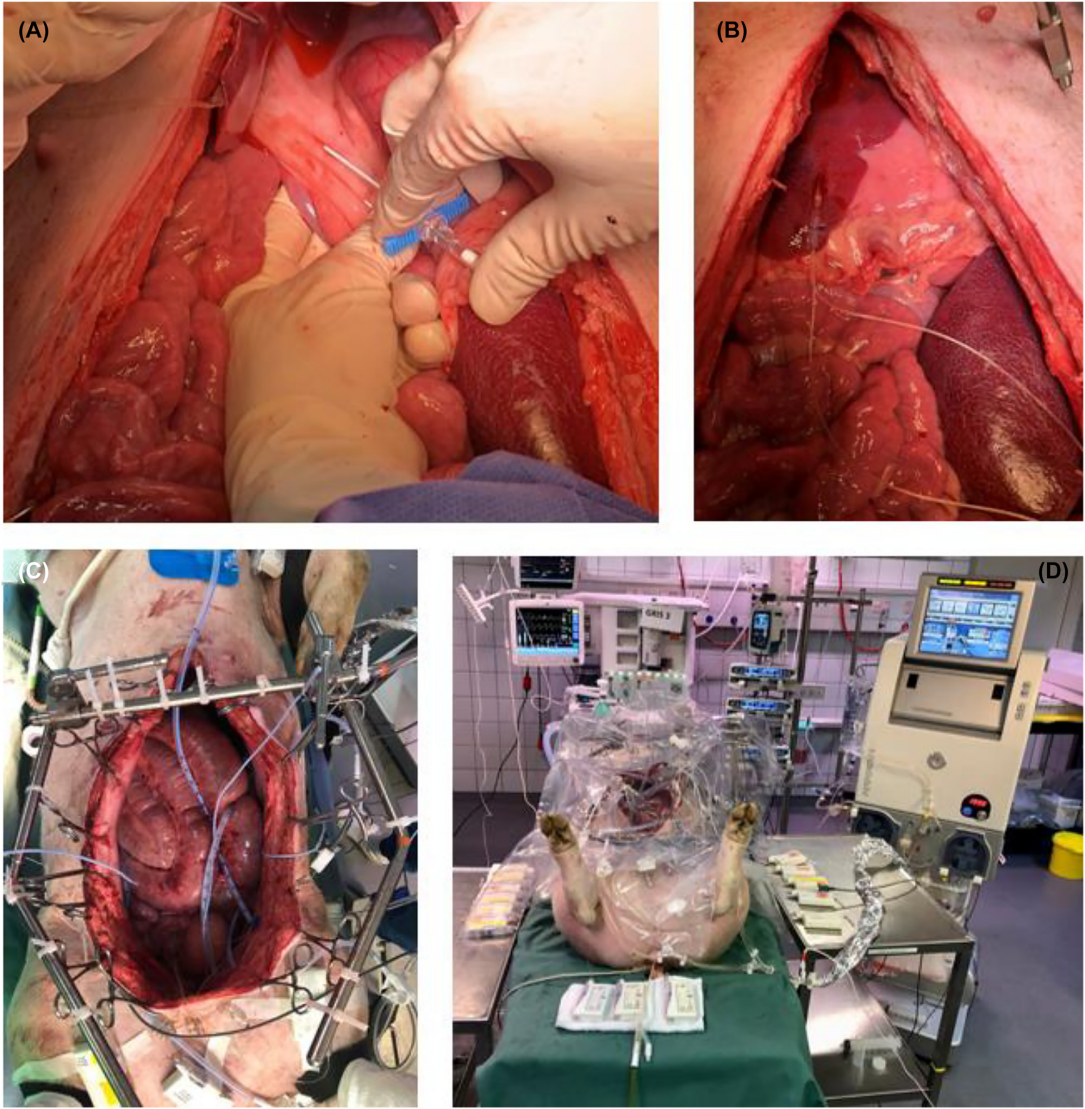
Illustrative example of placement of microdialysis catheter. (A) Illustrative example of placement of microdialysis catheter in hepatoduodenal ligament inserting an introducer. (B) Illustrative example of placement of a microdialysis catheter in the liver. The catheter is fastened to the liver tissue with a loose single suture to avoid displacement during sampling period. The same was done for all catheters. (C) Setup before HIPEC procedure seen from above. Placement of HIPEC tubes, HIPEC temperature probes and microdialysis catheters are shown. (D) Final setup placement of microdialysis pumps on tables, HIPEC performer system, and abdominal protection cover.

Eight microdialysis catheters were placed in various solid tissues after the surgical procedure (Table 1) (Figure 2A and B). In the rectum and the hepatoduodenal ligament, catheters were placed in two depths (one mm and four mm), verified with ultrasound. Placement depth was not measured for the other compartments but the catheters were placed as superficial as possible. Catheters in the rectum were placed in the anterior rectum wall in relation to the upper part of the Pouch of Douglas. After placement, the microdialysis catheters were perfused with 0.9% isotonic saline solution and 30 min tissue equilibration was allowed.

### HIPEC procedure

Initially 6 L of 0.9% NaCl solution was heated and used as carrier solution with 1 L as a reservoir in the pump system (Performer HT^®^ system from RAND, Medolla, Italy). Carrier solution volume of 6 L was opted due to the size of the abdominal cavity of the animals. Two inflow tubes were placed within the animal abdomen and three outflow tubes were placed via an incision in the abdominal wall near the liver, the spleen and in the pelvis. Temperature probes were attached to the outflow tubes to detect the temperature near the liver, the spleen and in the pelvis. A temperature probe was attached to the inflow tubes as well. When the intraabdominal temperature was stable at 41–42 °C, carboplatin 800 mg/m^2^ (10 mg/mL, Fresenius Kabi, Bad Homburg, Germany) was given as a single dose via the inflow tubes. Determination of body surface area for each pig was made with the following equation [21]:

$$\text{SA}=0.0970\cdot W^{0.633}$$

SA is the pig body surface area (m^2^) and *W*  is the body weight (kg). Based on this calculation, the carboplatin dosage range was 1,153–1,272 mg. Perfusion time was 90 min based on common clinic practice [22]. A plastic abdominal cover was placed over the setup to reduce heat loss (Figure 2C and D). Temperature within the abdomen was kept at 40–42 °C during perfusion. Immediately after the perfusion period the abdominal cavity was drained completely.

### Sampling procedure

Time zero of the 8-h sampling period was marked by HIPEC initiation. Dialysate samples were collected at 30 min intervals from time 0–240 min, and at 60 min intervals from time 240–480 min (Figure 1). A total of 12 samples were collected from each catheter. Following the sampling period, the microdialysis perfusate was replaced with a 0.9% NaCl solution containing 100 µg/mL carboplatin. Next, all catheters were allowed for 20 min equilibration, followed by two recovery samples at 40 min intervals. Blood samples were drawn from a central venous catheter in the midpoint of the microdialysis sampling intervals. Samples from the HIPEC perfusate were collected immediately after procedure completion to assess the concentration in the perfusate. Dialysate samples were stored immediately at −80 °C until analysis. Blood samples were stored for maximum 2 h at 5 °C until being centrifugated at 2,500 *g*, at 20 °C and for 10 min. Plasma aliquots were stored at −80 °C until analysis.

### Liquid chromatography tandem mass spectrometry (LC-MS/MS)

The free concentration of carboplatin in dialysates and plasma were analysed by an LC-MS/MS method. The analyses were conducted on a Waters Acquity ultra-performance liquid chromatograph (UPLC) with Xevo TQ-S tandem mass spectrometer operated in electrospray positive mode. The mobile phases consisted of 100 mM ammonium formate, 0.1% formic acid in water (mobile phase A) and Acetonitril (mobile phase B).

For analysis of carboplatin in dialysates, 5 µL dialysate, calibrator or control was mixed for 15 min at 37 °C using a Hamilton STARlet workstation, with 50 µL internal standard (2.0 μg/mL Carboplatin-d4 in ammonium formate 100 mM, 0.1% formic acid in water). Subsequently, the samples were precipitated by adding 365 µL acetonitrile followed by 4 min mixing at room temperature. After centrifugation for 20 min at 1,520 *g*, 250 µL of the supernatant was transferred to a new microtiter plate.

For analysis of carboplatin in plasma, samples were ultrafiltrated in Pall Corporaton multi-well filter plates. 300 µL plasma was pipetted on the Hamilton STARlet workstation in the plate and placed on top of a 700 µL collection plate for 10 min centrifugation at 1,520 *g*. The lower plate with the filtrate was then sample prepared as described for dialysates.

Four µL of the prepared samples were injected on a Waters Acquity UPLC^®^ BEH Amide, 1.7 µm column 2.1 × 50 mm. The initial conditions were 8% A for 1.0 min, linear gradient to 45% A at 1.7 min, and then the column was rinsed at 90% A from 1.8 to 2.7 min and finally re-equilibration at 8% S from 2.8 to 3.8 min. The liquid flow was 0.60 mL/min throughout. The needle and injector was cleaned between each injection by 400 µL strong wash (acetonitrile) and 1,200 µL weak wash (acetonitrile:water 1:9).

The multiple reaction monitoring (MRM) transitions used for carboplatin were 371.97>293.88 as quantifier and 371.97 > 354.87 as qualifier whereas the d4-Carboplatin was detected at 376.97 > 298.88 although the most abundant M+1 isotope for the d4-Carboplatin would be 375,97. The isotope 376.97 was chosen to avoid interference on 375.97 from a low abundant, unlabelled carboplatin isotope with the same mass that constitutes approximately 0.5% of carboplatin molecules.

The analysis was calibrated by in-house prepared calibrators (0–100 μg/mL) and the intermediate precision for the internal controls at five levels were 20.8% (target 0.100 μg/mL, n=18), 11.1% (target 1.00 μg/mL, n=18), 9.7% (target 4.00 μg/mL, n=18), 3.5% (target 30.0 μg/mL, n=6) and 6.8% (target 100.0 μg/mL, n=8). The lower limit of quantification (LOQ) was 0.100 μg/mL.

### Pharmacokinetics and statistical analysis

Pharmacokinetic data were determined by non-compartmental analysis using STATA (v. 17, StataCorp LLC, College Station, TX, USA) for all eight compartments in all animals. The pharmacokinetic parameters were peak drug concentration (C_max_), time to peak drug concentration (T_max_), area under the concentration-time curve from 0 to last sample collection (AUC_0-last_) and half-life (T_1/2_). C_max_ was calculated as the peak drug concentration during the sampling period, and T_max_ as the time to C_max_. AUC_0-last_ was calculated using the linear up-log down trapezoidal method. T_1/2_ was calculated as $\text{ln}\left(2\right)/\lambda_{\text{eq}}$ , where $\lambda_{\text{eq}}$ is the terminal elimination rate constant estimated by linear regression of the log concentration on time.

All parameters were analysed using a mixed model, repeating measures analysis of variance. Assumptions of normal distribution residuals and error terms were assessed by Quantile–Quantile plots. Assumption of homogeneity of the variance of error terms was assessed by residuals versus fits plots. Giving the small sample size a correction of freedom by the Kenward–Roger approximation method was used. Comparisons between compartments were assessed using F-test and paired t-test. Significance was considered when p<0.05.

## Results

All eight pigs completed the study. Malfunction of catheters was experienced for three of 64 catheters (one peritoneum and two rectum profound). Displacement of catheters outside the target tissue was observed for eight of 64 catheters (two rectum superficial, one rectum profound, five bladder). In total, samples were collected from 53 microdialysis catheters. Mean RRs for the individual compartment are shown in Table 2.

**Table 2:** Mean (SD) relative recoveries shown for all compartments.

Compartment	Liver	HL superficial	HL profound	Peritoneum	Rectum superficial	Rectum profound	Mesentery	Bladder wall
Mean (SD) relative recovery	89.5% (4.6)	91.0% (8.2)	88.1% (5.1)	75.3% (11.9)	93.1% (3.6)	87.0% (12.7)	75.2% (10.1)	78.7% (12.4)

HL, hepatoduodenal ligament.

The mean (SD) carboplatin concentration of the perfusate at the end of HIPEC was 278 µg/mL (62). The mean (SD) perfusate volume during HIPEC was 5,780 mL (707).

**Figure 3:**
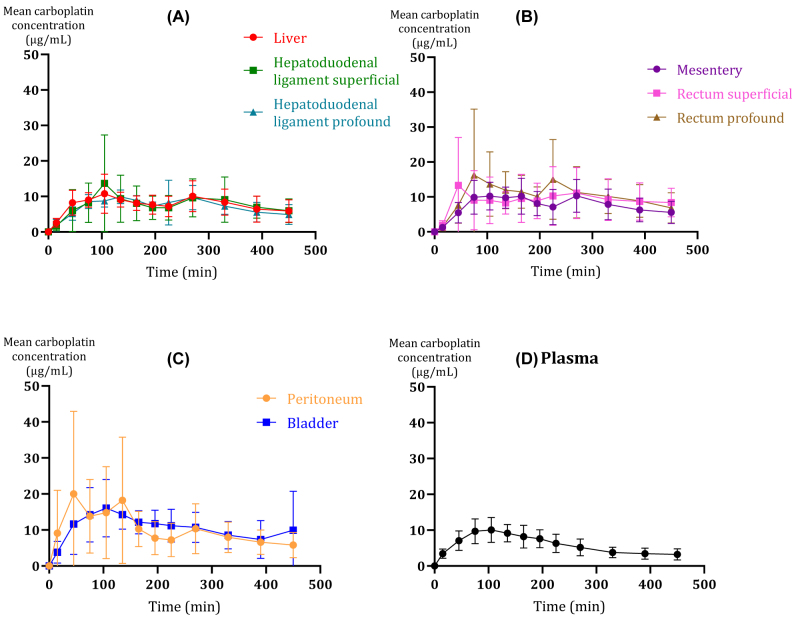
Mean concentration-time profiles with subdivision of compartments by anatomical regions. (A) liver, profound and superficial hepatoduodenal ligament (HL), (B) mesentery, rectum profound and superficial, (C) peritoneum and bladder wall, and (D) plasma. The bars represent 95% confidence interval.

Carboplatin was similarly distributed to peritoneal surfaces in the abdominal cavity evaluated by the mean AUC_0-last_ and C_max_ (Table 3 and Figure 3). No statistically significant differences in carboplatin AUC_0-last_ were found between the compartments, except for the bladder and the peritoneum, which expressed a higher AUC_0-last_ compared to plasma (p<0.05).

**Table 3:** Pharmacokinetic data for carboplatin given as mean (95% confidence interval) for all compartments.

Compartment	n	AUC_0-last_ (min·µg/mL)	C_max_ (µg/mL)	T_max_ (min)
Liver	8	3,469 (2,122; 4,816)	13.0 (4.6; 21.3)	206 (124; 289)
HL superficial	8	3,557 (2,210; 4,904)	15.4 (7.1; 23.8)	186 (103; 268)
HL profound	8	3,299 (1952; 4,646)	12.6 (4.3; 21.0)	178 (96; 261)
Peritoneum	7	4,561 (3,171; 5,951)	26.0 (17.2; 34.7)	196 (109; 283)
Rectum superficial	6	3,639 (2,192; 5,086)	15.1 (5.8; 24.3)	175 (81; 268)
Rectum profound	5	4,207 (2,683; 5,732)	20.6 (10.7; 30.5)	205 (103; 307)
Mesentery	8	3,300 (1953; 4,647)	12.4 (4.1; 20.8)	197 (114; 279)
Bladder wall	3	5,176 (3,367; 6,985)	22.0 (9.7; 34.2)	197 (69; 326)
Plasma	8	2,657 (1,310; 4,004)	10.6 (2.2; 19.0)	105 (23; 187)

AUC_0-last_, area under the curve from 0 to time for last sample collection; C_max_, peak drug concentration; T_max_, time to peak drug concentration; HL, hepatoduodenal ligament; n, number of animals from which samples were collected.

For the hepatoduodenal ligament, the mean AUC_0-last_ and C_max_ for the superficial catheter were higher than for the profound catheter (AUC_0-last_: 3,557 vs. 3299, and C_max_: 15.4 vs. 12.6 µg/mL). Contrary, in the rectum compartment, the mean AUC_0-last_ and peak drug concentration for the profound catheter were higher than for the superficial catheter (AUC_0-last_: 4,207 vs. 3638, and C_max_: 20.6 vs. 15.1 µg/mL). No statistically significant difference was found.

Within the investigated solid tissues, the lowest and highest mean C_max_ were found in the mesentery (12.4 µg/mL) and the peritoneum (26.0 g/mL), respectively. However, no significant differences were found for C_max_ among the investigated solid tissues. Only, mean plasma C_max_ (10.6 µg/mL) was found lower than peritoneum C_max_ (p<0.01).

T_max_ was comparable between all the compartments, with the lowest mean T_max_ in plasma (105 min), while the investigated solid tissues ranged from 175-206 min. At the last samples obtained (450 min) the mean carboplatin concentrations were 4.9–9.9 µg/mL across the investigated solid tissues.

## Discussion

To our knowledge, this is the first study to apply microdialysis for the assessment of *in vivo* carboplatin abdominal target tissue concentrations during and after HIPEC in a porcine model. In a recent review it is stated that clinically relevant preclinical models are warranted for studying HIPEC in a systematic way [23]. Furthermore, knowledge of tissue drug concentrations is essential for HIPEC anticancer efficacy evaluation. The main findings were similar distribution of carboplatin to all the investigated solid tissues together with comparable carboplatin concentrations at different penetration depths in rectum and the hepatoduodenal ligament. This novel porcine model allows for future studies of the individual HIPEC parameters such as temperature regulation, chemotherapeutic drug dosage and HIPEC duration, which are required to establish a fully validated procedure for clinical use.

The mean carboplatin concentration range was of 4.9–9.9 µg/mL across the investigated solid tissues at time 450 min. Interestingly, mean T_max_ for all compartments was longer than the 90 min HIPEC procedure. Plasma presented with the lowest mean T_max_ (105 min), while most solid tissues displayed a T_max_ of 180 min or longer which may indicate a noteworthy long time for local equilibrium. Previous studies report sparse peritoneal carboplatin clearance and a long terminal half-life of the perfusate, and combined with the fact that it is difficult to empty the abdominal cavity completely and some of the perfusate might be left in the cavity, this may explain the prolonged T_max_ and the high concentrations in the end of the sampling period [14, 24].

It is complex to evaluate tumour cell eradication potential based on tissue concentrations. A few studies suggest the half-maximal inhibitory concentration (IC50) or 50% growth inhibition concentration (GI50) as suitable markers of efficacy. IC50 values and GI50 values differ with each cancer cell line and for e.g. ovarian cancer, up to 100 cell lines exists [25]. In cancer research, cancer cell lines have on several occasions been applied in *in vitro* models to study treatment strategies, but it is problematic and difficult to translate *in vitro* experiment/model results to *in vivo* studies/observations. Although not significant different, the presented carboplatin tissue C_max_ concentrations varied somewhat across the investigated solid compartments. Peritoneum presented with the highest C_max_ (26 µg/mL), while the liver (13.0 µg/mL) and the mesentery (12.4 µg/mL) demonstrated the lowest C_max_. Accordingly, not all tissues reached a relevant range of theoretical IC50 values, ranging from 51.3 µM (19 µg/mL) to 1.66 × 10^8^ µM (62 mg/mL) [26].

Presence of residual tumour nodules measuring 2.5 mm in diameter or smaller has been defined as optimal surgery [4]. Therefore, the secondary aim was to investigate, whether penetration depth of carboplatin in different tissues could be assessed by microdialysis, but more importantly to assess whether carboplatin during HIPEC reaches deeper tissue penetration than only reaching the surface. Our results may suggest that carboplatin penetrates to a minimum depth of four mm., but it could be speculated, whether the carboplatin concentrations in the profound compartments represent a systemic drug delivery or a local, especially in the profound rectum compartment which unexpectedly showed higher concentrations than in the superficial compartment. Carboplatin abdominal tissue concentrations and penetration depths have previously been studied by tissue biopsies in a tumour rat model [6] and in tumour mouse model [7], following intraperitoneally carboplatin bolus injections. Although we applied a translational HIPEC setting in contrast to intraperitoneal bolus injections, the results from the rodent studies are in line with our findings suggesting sufficient abdominal tissue penetration regarding tissue depths for carboplatin. For future microdialysis studies it is, however, important to further explore carboplatin penetration depth properties.

For plasma, mean carboplatin AUC_0-last_ and C_max_ tended to be lower than for all other investigated solid tissues. Mikkelsen et al. [14] studied plasma and perfusate carboplatin pharmacokinetics during HIPEC in ovarian cancer patients, and report mean plasma AUC_0-90min_ of 1,926 min·µg/mL and mean plasma C_max_ of 29 µg/mL. In the present porcine study, a mean plasma AUC_0-last_ of 2,657 min·µg/mL and a mean C_max_ of 10.6 µg/mL was found, which is above previous reported toxicity threshold steady-state concentrations [27]. Mean carboplatin plasma AUC_0-last_ did not exceed reported levels associated with toxicity [15]. Several factors could reason the different findings between the present study and the study by Mikkelsen et al.; First, all eight pigs were young and healthy. Furthermore, the CRS performed in this study, may not fully represent a clinical setting after extensive cancer surgery with regards to procedures, operation time and bleeding. Moreover, tumour tissue and normal tissue differ with several characteristics [28]. Chemotherapeutic drug delivery to tumour tissue has been widely studied, suggesting that penetration is inhibited by various tumour tissue characteristics in comparison to normal tissue, such as abnormal vascular structure and elevated interstitial fluid pressure [29]. Although pigs and humans share many similarities in anatomy and physiology, these aspects are important to note when considering the translational potential of the present data [30, 31]. Second, perfusate volume was 6,000 mL in the present study, in comparison to 5,000 mL in the study by Mikkelsen et al., due to larger abdominal cavities of the pigs. In clinical settings, no standard recommendation regarding volume of the carrier solution has yet been compiled and protocols vary a lot [32], but most centres use 2 L/m^2^ corresponding to approximately 4,000 mL for a human [22]. Despite different perfusate volumes, our mean carboplatin concentration of the perfusate at the end of HIPEC (278·µg/mL) was comparable to the findings by Mikkelsen et al. Therefore, the carboplatin dosage in the present study setup is believed to be compliant to the size of the abdominal cavity. However, it is possible that the animals received a smaller total dosage of carboplatin and for future experimental HIPEC studies, smaller animals may be considered. Moreover, we employed a rather low flowrate (750 mL/min) of perfusate circulation during HIPEC and two inflow and three outflow tubes. Flowrate is not reported in the study by Mikkelsen et al. In clinical settings three inflow and two outflow tubes, and higher flow rates (>2000 mL/min) are normally used [33, 34]. Despite these differences, similar drug distribution and an outflow temperature during HIPEC>40 °C indicates sufficient HIPEC perfusate circulation within the abdominal cavity.

Regarding microdialysis, it is important to consider certain limitations especially in relation to RR [20]. Precautions were taken to prevent variations related to RR in our study: First, a low flow rate of 1 µL/min perfusate was used; second, long membranes of the microdialysis catheters were applied; third, adequately sampling intervals were chosen to collect enough dialysate for analysis. Although the microdialysis method possess many advantages, no gold standard exists to validate our findings.

In conclusion, microdialysis was successfully applied for the assessment of carboplatin concentrations in various abdominal target tissues during and after HIPEC. The main findings were similar distribution of carboplatin, detection of carboplatin at a minimum of 6 h after HIPEC completion and a carboplatin penetration depth of minimum four mm. Future studies may benefit from this feasible and novel porcine model and such studies are required for testing interventions, setups and anticancer efficacy.
